# Improving Temporal Consistency of Preferences: The Influence of Mental Construal

**DOI:** 10.5964/ejop.v14i4.1379

**Published:** 2018-11-30

**Authors:** Asli Elif Aydin

**Affiliations:** a School of Applied Sciences, Istanbul Bilgi University, Istanbul, Turkey; Department of Psychology, Webster University Geneva, Geneva, Switzerland; Department of Psychology, School of Social Sciences, Heriot Watt University, Edinburgh, United Kingdom

**Keywords:** preference consistency, construal level theory, mental representations, psychological distance, electronic products

## Abstract

Majority of the current literature on mental construal has focused on effects of varying construal levels on preference shifts whereas this research investigates the influence of mental construal on the change of preference consistency over time. Building on construal level theory, we propose that high-level construal, which creates abstract, and decontextualized mental representations, leads individuals to more consistent preferences than low-level construal, which creates concrete, and contextualized mental representations. Furthermore we examine the effect of having a matching versus non-matching construal level at two different evaluation instances, on achieving greater extents of consistency. To test this prediction a mixed experimental design is employed, in which participants evaluated electronic products at two different sessions. It is demonstrated that when participants have the same construal level at two points in time, their evaluations become similar since they mentally construe the objects in the same way whereas when the construal level differs at these two points, participants focus on different aspects of the products, form different evaluations and have less consistent preferences.

Prior research supports the notion that individuals are motivated to be consistent (Fishbach, Ratner, & Zhang, 2011). Several theories within psychology literature demonstrate the inherent struggle of people to achieve consistency of beliefs, attitudes and behaviors (e.g. Balance theory, Heider, 1946; Cognitive dissonance theory, Festinger, 1957). These theories recognize that consistency seeking is a strong urge, which controls individuals’ behavior (Cialdini, 2009). Through creating tension in one’s state of mind, inconsistencies motivate individuals to seek out ways to restore a stable equilibrium. Nonetheless, consistency is hard to achieve. The constructed nature of preferences, indicating that preferences are created whenever individuals faced a decision problem, hinders individuals’ ability to be consistent in their preferences (Lichtenstein & Slovic, 2006). Even when individuals are satisfied with a previous choice, they may fail to be consistent due to numerous factors related to the task, choice context and characteristics of the decision maker (Payne, Bettman, & Johnson, 1992).

A valuable framework to recognize variations in individuals’ preferences comes from the construal level theory (CLT), which suggests that when people mentally construe objects at a high level, the fundamental characteristics of the object are more discernible, yet if they construe objects at a low level the details become more salient (Trope & Liberman, 2000). While past studies (Chang & Liu, 2008; Dhar, Nowlis, & Sherman, 2000; Hsee, Yang, Gu, & Chen, 2009; Mishra, 2009) have mostly focused on the impact of mental construal on the types of information people selectively attend to in evaluating alternatives and forming preferences, the current study adopts a different perspective. We focus on examining the change in the degree of consistency at different construal levels.

There has been only limited number of studies that have investigated the relationship between construal levels and degree of preference consistency and they have come up with conflicting results (e.g. Fukukura, Ferguson, & Fujita, 2013; Khan, Zhu, & Kalra, 2011; Van Kerckhove, Geuens, & Vermeir, 2015). From a theoretical standpoint it is essential to add to the scarce amount of research in this stream and provide support to either one side of the argument, in this case favoring the increased consistency of preferences with high level construal. Furthermore, it is also imperative to reveal the type of mindset that will induce more consistent preferences from a practical standpoint. It has been demonstrated that decline in consistency of preferences leads to regret and dissatisfaction with choice (Lee & Zhao, 2014). Therefore, understanding this relationship and providing individuals with tools and practices that will reduce inconsistencies and negate the adverse consequences will be valuable to attain contented individuals.

Through this study, an important contribution to the literature is made by shedding light on the impact of change in mental construal on the degree of preference consistency. In doing so, we employ a temporal measure of preference consistency which enables us to investigate the effect of having a matching versus a non-matching construal at two different evaluation instances on the degree of preference consistency. Thus, we also contribute to the literature by demonstrating how changing the construal level impacts preference consistency over time. This inquiry is valuable considering the variable nature of construal levels with situational factors.

Consequently, drawing from construal level theory it is suggested that high-level construal, which creates abstract, global, and decontextualized mental representations, lead individuals to more consistent preferences than low-level construal, which creates concrete, local, and contextualized mental representations (Trope & Liberman, 2010). Additionally, we look into the impact of the match of construal levels at different evaluations on the degree of preference consistency. By means of this, it is suggested that matching mindsets at two distinct points of evaluation should produce more consistent preferences than non-matching mindsets.

This paper is organized as follows. First, the theoretical background on the construal level theory is reviewed. Moreover the impact of mental construal on the change of preference consistency is outlined and hypotheses are developed. Following that the method of the study is explained. Next, the data is analyzed and the results of the study are demonstrated. Finally, the findings are discussed together with their theoretical and practical implications. Limitations of the study and directions for future research are also provided.

## Theoretical Background

### Construal Level Theory

Construal level theory (CLT, Liberman & Trope, 2008; Trope & Liberman, 2000) suggests that every behavior can be mentally represented in an abstract or a concrete manner. High-level construal is associated with abstract mindsets, yet low-level construal is associated with concrete mindsets (Freitas, Gollwitzer, & Trope, 2004; Trope, Liberman, & Wakslak, 2007). Construal level increases with psychological distance (e.g. temporal distance, spatial distance, social distance and hypotheticality), which causes more abstract mental representation of objects (Liberman, Trope, & Wakslak, 2007; Trope & Liberman, 2003). Besides, construal level can also be primed. It is stated that depending on the focus on how the behavior is performed or why it is performed, the level of mental abstraction can be shifted from low-level end to high-level end of construal continuum (Vallacher & Wegner, 1987; Vallacher & Wegner, 1989).

CLT is mainly related to the type of information that is selectively put to use while making an evaluation (Lynch & Zauberman, 2007). A number of studies attempted to sort out the characteristics, which receive varying weights in evaluations depending on the mental construal. One such characteristic is primary versus secondary nature of product attributes (Kray, 2000; Liviatan, Trope, & Liberman, 2008a). It is demonstrated that core features of objects (e.g. sound quality of a radio) have a more significant role in judgment than peripheral features of objects (e.g. timer of a radio) when construal level is high than when it is low (Lee, Deng, Unnava, & Fujita, 2014; Torelli & Kaikati, 2009; Trope & Liberman, 2000). Likewise, it is indicated that benefit based appeals are more persuasive than attribute based appeals when construal level is high than when it is low (Hernandez, Wright, & Ferminiano Rodrigues, 2015).

A further characteristic of construal levels is desirability versus feasibility aspects. Desirability aspects refer to end state while feasibility aspects refer to means used to reach that end state (Liberman & Trope, 1998; Sagristano, Trope, & Liberman, 2002). It is shown that high desirability and low feasibility options (e.g. getting 10 free CDs as a promotion at an inconvenient location) are preferred to low desirability and high feasibility options (e.g. getting 1 free CD as a promotion at a convenient location) when construal level is high than when it is low (Todorov, Goren, & Trope, 2007). Additionally, it is determined that desirability aspects of an object have a stronger effect on purchase intentions for the distant future and feasibility aspects of an object have a stronger effect on purchase intentions for the near future (Thomas, Chandran, & Trope, 2006). Gift giving context displayed equivalent results. It is demonstrated that compared to the receivers of the gift, gift givers put more weight on desirability aspects and less weight on feasibility aspects as a result of their’ increased psychological distance (Baskin, Wakslak, Trope, & Novemsky, 2014).

Attribute alignability constitutes an additional varying characteristic between levels of construal. Since non-alignable attributes, which have no corresponding features in the other alternative, require an abstract mental representation, they are emphasized more with high-level construal. Likewise, alignable attributes, which are comparable features with the other alternative requires a concrete representation; hence they are emphasized more with low-level construal (Pfeiffer et al., 2014; Trope & Liberman, 2010). Consequently, alignable better alternatives are preferred when construal level is low and non-alignable better alternatives are preferred when construal level is high (Malkoc, Zauberman, & Ulu, 2005).

Overall, as indicated in these sample studies, when construal level is high evaluations focus on abstract, decontextualized, core features and desirability concerns; and when construal level is low evaluations focus on concrete, contextualized, peripheral features and feasibility concerns (Ledgerwood, Trope, & Chaiken, 2010).

### Relationship Between Construal Level and Change in Preference Consistency

As summarized above, an ample body of research on CLT examined how changes in construal level shift individuals’ preferences. These studies focused on the influence of mental construal on the importance given to different types of attributes in evaluations (Lynch & Zauberman, 2007). However a related field of research investigating the extent of change in preference consistency between individuals with high versus low level construal levels received very limited attention. On top of that, those few studies that examined how mental construal influences the degree of preference consistency presented conflicting results.

For instance, Kardes, Cronley, and Kim (2006), suggested that preference formed on the basis of lower construal level should be more stable over time. In particular, it was indicated that stability of preferences increased in low-level construal as vividness and concrete details of the experimental stimuli ease preference formation process. It is indicated that since large amount of contextual detail create greater confidence in judgments (Gill, Swann, & Silvera, 1998), and preferences made based on contextual details are more resistant to persuasion (Kallgren & Wood, 1986), a concrete mental representation of objects should produce more consistent preferences.

In accordance with that Van Kerckhove et al. (2015) proposed that looking down to process proximal stimuli, which is connected with low construal levels, creates more consistent preferences than looking up to process distant stimuli, which is connected with high construal levels. Based on their preliminary findings it was argued that high construal levels induce construction of preferences instead of recall of initial preferences since a varying set of choice related information is incorporated in decisions.

Contrary to that, Khan et al. (2011) reported mixed findings regarding the effect of mental representations on context dependent trade-offs. Specifically, in deliberative comparisons, context effects such as the compromise, and the background-contrast effects, produced decreased number of trade-offs with abstract mindsets compared to concrete mindsets. In other words, preferences were more stable with abstract mindsets regardless of the choice context. It is suggested that in effortful processing high-level construal shift attention away from detailed trade-offs; thus shield preferences from contextual effects. However, for the attraction effect, which emerges on account of intuitive processing, the opposite pattern is observed.

Earlier studies that suggest increased preference consistency with low-level construal employ overly simplistic stimuli that lack contextual details and don’t necessitate effortful processing. However for the majority of the choice problems, the nature of the alternatives and the decision circumstances are considerably more complex and detailed. Further it is established that when there is a lot of information, abstraction improves decision outcome in terms of selecting the better alternative (Fukukura et al., 2013).

In the current study, it is expected that consistency of preferences increase when decisions are made with an abstract mindset rather than a concrete mindset. It is stated that high-level mental representations are more coherent than low-level mental representations (Liberman, Sagristano, & Trope, 2002). Furthermore, it is suggested that high-level mental representations reflect central features and omit incidental features. At a close distance representations highlight contextual details, yet as distance increases, representations convey the substance of the target object. These high-level features enable general evaluations that encapsulate constant aspects of a target object across multiple context, thus preferences will become more consistent (Ledgerwood, Wakslak, & Wang, 2010). Therefore it is proposed that:

*Hypothesis 1:* A high-level construal produces more consistent preferences than a low-level construal.

Above-mentioned studies all acknowledged that it is possible for an individual to adopt a different mental construal when faced with a previously encountered choice task and result in a change of preference due to the difference in individual’s mindset. Yet, most studies employed an operationalization of consistency which is assessed by comparing the preferences of individuals in different treatment groups (Lynch & Zauberman, 2007). Testing dynamic consistency of the same individuals with different construal levels in time has been a virgin territory in this stream of research. It is expected that repeated measures will present novel findings for construal studies.

No prior work in CLT literature, to the best of the author’s knowledge, has employed a procedure that enables to evaluate preference consistency at a temporal level. In just one conceptual work, Kardes et al. (2006) predicted that evaluations would be more stable over time when construal levels at two points in time are similar. However, this conjecture has not been empirically confirmed. Without using a different methodology to measure consistency of preferences across varying construals levels, it will not be possible to investigate the effect of having a matching vs. non-matching mental construal on the preferences of the same individual.

Similar to what Kardes et al. (2006) suggested, it is expected that dynamic preferences will be more consistent in time when construal levels at two evaluation instances in time match. The main reasoning for this assertion is that same cues will be discernible at choice and retrieval. In particular, when individuals adopt a high-level mental construal at both instances of evaluation, they focus on same characteristics namely, core, primary features, and desirability aspects. Consequently, their preferences will be consistent. On the other hand, if they adopt a low-level mental construal at the second evaluation, after adopting a high-level mental construal at the initial evaluation, this time they focus on different characteristics namely, peripheral, secondary features, and feasibility aspects. Thus, their preferences will be inconsistent. As a result, it is suggested that preferences will be more consistent when the levels of mental construal at two points in time (T1 & T2) are matching than when they are non-matching. In other words, when the level of construal of the first evaluation matches that of second, preferences will be more consistent. Accordingly it is proposed that:

*Hypothesis 2a:* Preferences will be more consistent when a high-level construal at T1 is followed by a matching high-level construal rather than a non-matching low-level construal at T2.

*Hypothesis 2b:* Preferences will be more consistent when a low-level construal at T1 is followed by a matching low-level construal rather than a non-matching high-level construal at T2.

## Method

### Participants and Design

The study took place at two universities where subjects were given extra course credits to participate. Since the study had a within subject design with two sessions, the responses of only those who participated in both sessions were analyzed. Of the 235 who participated in the first part of the study, 172 subjects attended the second part (%73.2). Fourteen responses were eliminated since subjects did not accurately follow the instructions. The final sample consisted of 158 students. The sample consisted of third and fourth year undergraduate students with business major. 49% of the sample was female.

A 2 (construal T1: high vs. low) x 2 (construal T2: high vs. low) x 5 (product category: laptop, mobile phone, digital camera, tablet, e-reader) mixed between – within subjects quasi experiment design was employed.

### Experimental Procedure

The study was conducted in two sessions that were two weeks apart. Both sessions consisted of paper-and-pencil tasks, which took around 30 minutes to complete. Students were asked to write down a code with a minimum of 6 digits on their papers. This code is later used to collate the two separate completed questionnaires of the participants. In the first session participants initially completed a thought exercise for the mental construal manipulation task (Freitas et al., 2004; Hamilton & Thompson, 2007). They were asked to focus and elaborate on “improving and maintaining health” activity for this exercise. Those in the abstract (concrete) mental construal condition first read a passage about life goals (ways of achieving life goals), which explained why (how) people do things, they do. Next, they were asked to provide three reasons for engaging in (three actions to reach) “improving and maintaining health” activity (goal) and rate the importance of each given reason. Following that they were asked to complete a diagram in which they came up with successive higher-level reasons (lower-level means) for “improving and maintaining health”; hence thought progressively abstractly (concretely).

**Figure 1:**
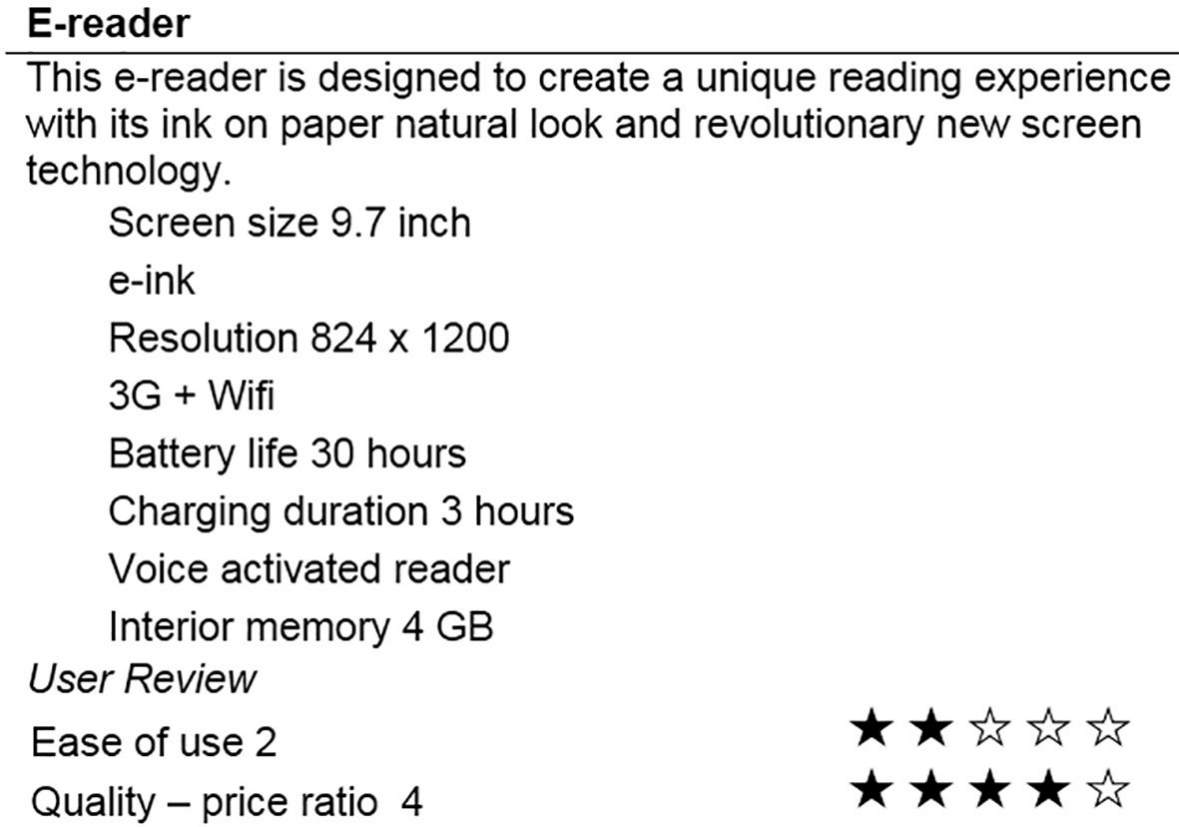
Sample stimulus.

In the next part, respondents were instructed to examine the descriptions of several electronic products, namely mobile phones, laptop computers, tablets, digital cameras, and e-readers and evaluate these products. Electronic products were chosen as experimental stimuli for two main reasons. First, electronic products, especially the ones used in this study, have a significant place in the lives of the university students hence they are frequently exposed to these products and information about them. Second, electronic products have adequate number of features that are all noticed and taken into account in choice process. Due to the temporal nature of the study it is important to find products that have sufficient complexity in order to reduce memory effects. The products that are used in the study are chosen with a pretest. 35 student participants were asked to rate their level of involvement and familiarity with 10 electronic products and the five highest ranking products are selected for the study. For all five electronic products, eight product features were provided. Some of the features were primary features (e.g. processor speed for a laptop) while others were secondary (e.g. smart cover for a laptop). A brief statement of product description, indicating the core benefit or essential characteristics, was mentioned as well. Furthermore, customer ratings for ease of use and perceived value, specifically price-quality ratio, were presented on a five-point scale. Ease of use represented feasibility aspect of the products while perceived value represented desirability aspect of the products (See example stimulus in Figure 1). Afterwards, participants evaluated product alternatives on a fifteen-point scale ranged from 1 (very negative) to 15 (very positive) as a measure of their preferences. In addition, participants responded to two questions that measured (a) their involvement in purchase decision: How important would it be to you to make a right choice of this product? (b) familiarity: How familiar are you with this product category? Responses to these questions were all anchored on scales of 1 to 7 (not important at all- very important, not familiar at all- very familiar).

In the second session of the study, a different mental construal manipulation task was used (Liberman, Trope, McCrae, & Sherman, 2007). Participants were informed that the study was about story building with the intention to investigate how people interpret and form impressions of different events. They were instructed to visualize each event and respond to following questions. Six brief descriptions of actions were presented to participants in both conditions. Participants in the abstract mental construal condition were asked *why* the events could have happened, whereas those in the concrete mental construal condition were asked *how* the events could have happened.

In the next part, respondents were instructed to examine the product descriptions and evaluate the products again on a fifteen-point scale ranged from 1 (very negative) to 15 (very positive). Preference inconsistency was measured as the absolute difference between the ratings of the first and the second sessions. A score of zero indicated totally consistent preferences whereas a score of fourteen indicated totally inconsistent preferences.

Participants evaluated ten products, specifically two alternatives for each of the five product categories, in both sessions. Only one set of the alternatives was asked to be evaluated at both sessions. The other alternatives were placed to disguise the purpose of the study and prevent memory effects. The analysis utilized the ratings of the five common alternatives that were used in both sessions.

## Results

First, participants’ level of involvement, and familiarity for all product categories were examined. The level of involvement was highest for laptops and lowest for e-readers (*M*_laptop_ = 6.57, *SD*_laptop_ = 0.91; *M*_mobile_ = 6.29, *SD*_mobile_ = 1.17; *M*_tablet_ = 5.62, *SD*_tablet_ = 1.60; *M*_camera_ = 5.53, *SD*_camera_ = 1.42; *M*_e-reader_ = 3.72, *SD*_e-reader_ = 2.02). Similarly, participants were most familiar with laptops and they were least familiar with e-readers (*M*_laptop_ = 6.32, *SD*_laptop_ = 1.03; *M*_mobile_ = 6.29, *SD*_mobile_ = 1.01; *M*_camera_ = 5.06, *SD*_camera_ = 1.70; *M*_tablet_ = 4.59, *SD*_tablet_ = 1.85; *M*_e-reader_ = 2.96, *SD*_e-reader_ = 1.81).

Next, a 2 (construal T1) x 2 (construal T2) x 5 (product category) repeated-measure ANOVA is conducted to examine the overall effects of construal on preference consistency for different product categories. The analysis revealed a significant main effect of construal level at T1, *F*(1, 153) = 5.06, *p* = .03, η^2^_p_ = .03, indicating that participants, who were in the high-level construal condition at T1, were more consistent in their evaluations regardless of their construal mindset at T2 of the study. A similar kind of effect is observed for the main effect of construal level at T2, yet it was not significant, *F*(1, 153) = 2.74, *p* = .10, η^2^_p_ = .02. More importantly, as predicted, the interaction between construal levels at T1 and T2 was also significant, *F*(1,153) = 6.32, *p* = .01, η^2^_p_ = .04. These findings held when controlling for participants’ sex.

To break down the interaction between construal levels at T1 and T2, planned contrasts were performed comparing consistency levels for varying construal levels at T1 and T2. In Hypothesis1 it was expected that high construal levels would produce more consistent preferences than low construal levels. As expected, the degree of inconsistency was lower for participants, who were in high-level construal conditions for both sessions (*M* = 2.01) compared to those, who were in low-level construal conditions for both sessions (*M* = 2.71, *t*(53.05) = -3.28, *p* < .01). Therefore, Hypothesis 1 was supported (see Figure 2a). Furthermore, the analysis revealed a significant main effect of product type on preference consistency, *F*(4, 150) = 7.48, *p* < .001, η^2^_p_ = .17) indicating a change of consistency levels across product categories (see Figure 2b).

**Figure 2a:**
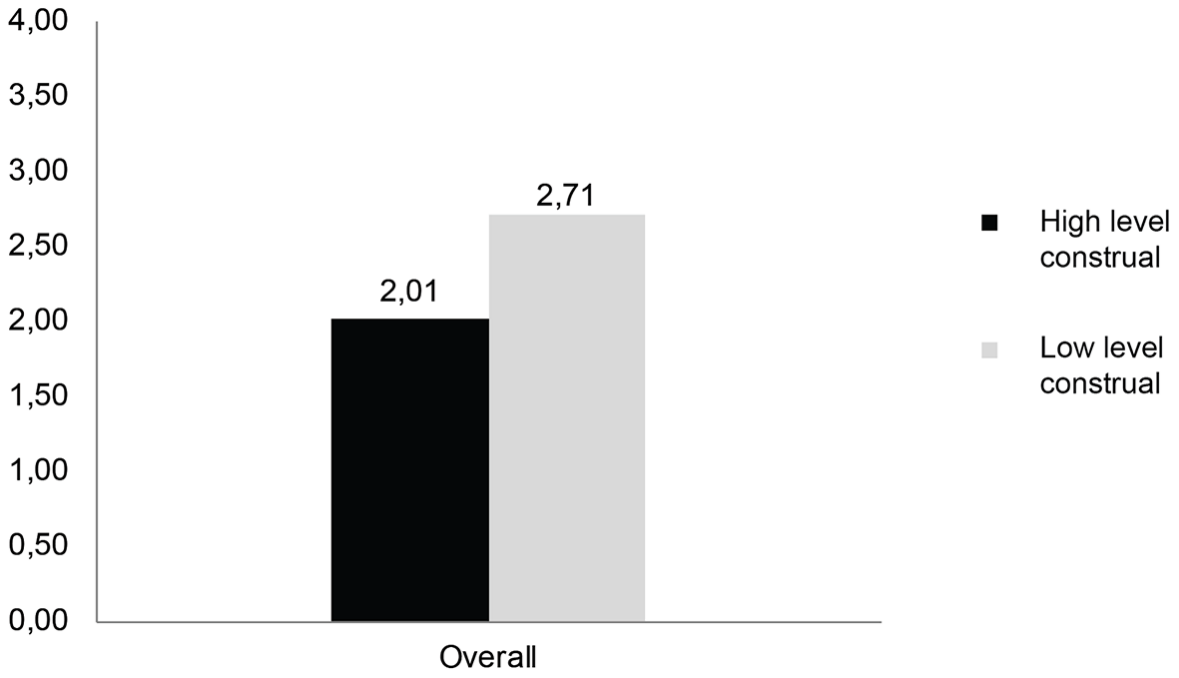
Overall mean preference inconsistency as a function of construal levels.

**Figure 2b:**
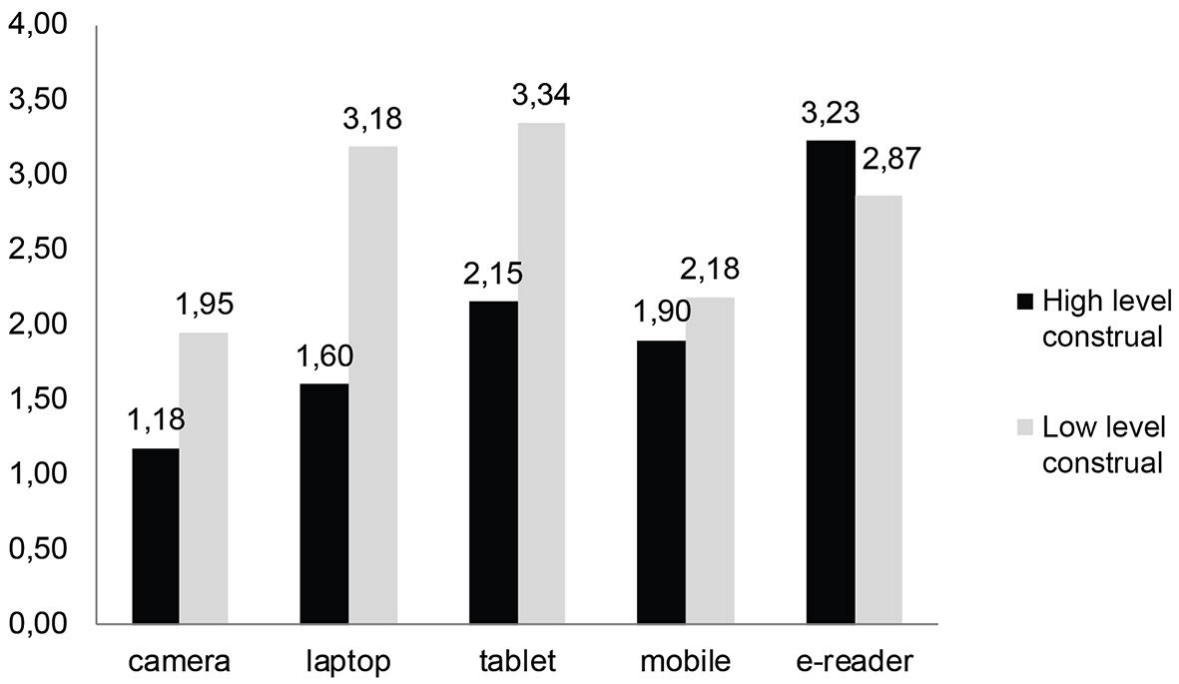
Mean preference inconsistency as a function of construal levels across product categories.

The interaction effect of construal level at T1 and product type was also significant, *F*(4, 150) = 7.48, *p* < .001, η^2^_p_ = .17. Other than that the interaction between construal level at T2 and product type, *F*(4, 150) = 1.05, *p* > .10, η^2^_p_ = .03, and triple interaction among product type, T1 and T2 construal levels, *F*(4, 150) = 0.20, *p* > .10, η^2^_p_ = .01, were not significant.

In the second hypothesis, it was expected that when the levels of mental construal at two points in time are similar, preferences would be more consistent than when they are different. Planned contrasts revealed that the level of inconsistency decreased significantly if a high-level construal at first evaluation is followed by a high-level construal (*M* = 2.01) than a low-level (*M* = 2.75) construal at second evaluation, *t*(51.99) = -3.39, *p* < .01. Therefore, Hypothesis 2a was supported.

Similarly, the level of inconsistency decreased significantly if a low-level construal at first evaluation is followed by a low-level construal (*M* = 2.71) than a high-level construal (*M* = 2.86) at second evaluation, however this effect was not significant, *t*(76.9) = -.544, *p* > .10. Therefore, Hypothesis 2b was not supported.

Following that, the same planned contrasts were run for each product category separately (Table 1). For laptop, tablet and digital camera results were parallel to overall analysis. For the mobile phone category, the findings were also similar yet the effect was not significant. For the e-reader, on the other hand, results with an opposite pattern were observed. The difference of preference inconsistency between high-level construal condition at both T1 and T2 and low-level construal condition at both T1 and T2 was not significant. Moreover, the difference between low-level construal condition at both T1 and T2 and low-level construal condition at T1 followed by a high-level construal condition at T2 was marginally significant.

**Table 1:** Contrasts of Preference Inconsistency for Matching Versus Non-Matching Mental Construal Levels Across Product Categories

Product Categories	Matching Construals	Non-Matching Construals	*t*
H2a (High construal at T1)				
Overall	2.010	2.750	3.394**
Digital camera	1.175	1.947	2.087*
Laptop	1.600	2.974	2.997**
Tablet	2.150	3.316	2.303*
Mobile Phone	1.900	2.615	1.556^†^
E-reader	3.225	2.932	0.490
H2b (Low construal at T1)				
Overall	2.710	2.860	0.544
Digital camera	1.949	2.561	1.135
Laptop	3.184	2.854	0.720
Tablet	3.342	2.732	1.213
Mobile Phone	2.184	2.268	0.182
E-reader	2.868	3.878	1.638^†^

^†^*p* < .10. *p < .05. **p < .01.

## Discussion

This study provides support for the hypothesis that individuals with high construal levels are more consistent in their evaluations than individuals with low construal levels. It is indicated that when construal level is high, individuals adopt an abstract mindset and when it is low they adopt a concrete mindset (Trope & Liberman, 2000). With the abstract mindset, individuals mainly focus on overall attractiveness of the objects and primary features addressing desirability concerns (Ledgerwood, Trope, & Chaiken, 2010). It is our prediction that abstract construal lead individuals to see the big picture and form global evaluations that remain intact. As the evaluations are made in a decontextualized manner, the essence of the evaluations remains the same instituting consistency. On the other hand, with the concrete mindset individuals focus on incidental aspects and peripheral features addressing feasibility concerns during evaluations. Individuals turn their attention to secondary aspects and stray into the jungle of details. Since the evaluations are made in a decontextualized manner in this case; they become inconsistent. This finding is validated across an assortment of product categories, namely digital camera, tablet, laptop, and mobile phone.

An exception to this finding is observed for a relatively low involvement / low familiarity product category. For such a product, the e-reader in this case, preferences are more consistent when individuals adopt a low-level mental construal than when they adopt a high-level mental construal. This finding is also in line with findings of earlier studies that utilized low involvement products (e.g. Kardes et al., 2006; Van Kerckhove et al., 2015). One probable account for this finding is that, with abstract representations concrete aspects of objects become vague and evaluations are made mostly based on information previously stored in memory (Bar-Anan, Liberman, & Trope, 2006). Individuals, who are not much familiar with a product category, find it more difficult to form general evaluations since they do not have sufficient prior knowledge structures (Bettman & Park, 1980). They cannot identify relevant and important information and instead they base their decisions on easily understandable features (Alba & Hutchinson, 1987). Consequently evaluations based on abstract representation lack a solid foundation and become unstable. On the other hand, evaluations based on concrete representations use heuristic cues that can be easily retrieved in following evaluations; hence relatively be more stable.

Another possible explanation for this result can be inferred based on action identification theory (Vallacher & Wegner, 1987). It is stated that when an activity is considered difficult to conduct as a result of lack of experience, individuals tend to focus on low-level details of the objects (Wan & Rucker, 2013). Consequently, for those in the low-level construal condition the primed mindset were congruent with the focused information, hence performance improved resulting in more consistent preferences.

Another important finding of the present study is that having matching construals at two points at time produces more consistent preferences than having non-matching construals. This pattern exists both for high-level construals and low-level construals, yet statistically significant only for high-level construals. It is our prediction that with matching mental construals, individuals focus on similar aspects of the objects and consequently evaluations become more consistent. Specifically, participants that are in high-level construal condition at both sessions pay more attention to similar primary features, and desirability concerns; therefore their evaluations at two sessions are parallel. Contrary to that, participants that are in high-level construal condition in the first and low-level construal condition in the second session form their opinions based on different product attributes due to non-matching mental representations; therefore their evaluations are not as consistent. For instance, a participant that is trying to assess a digital camera with an abstract mindset in the first evaluation may attend to the primary characteristics such as picture resolution and optical zoom. Following that, if a concrete mindset is adopted in the second evaluation, participants may focus on secondary attributes such as vibration reduction and Bluetooth connections. This may result in change in preferences causing inconsistencies.

For low construal levels the matching hypothesis turned out to be not significant. One probable account for lack of significance is that with a high-level construal, individuals initially see the abstract representation and following that with a low-level construal they form their evaluations on concrete details that they did not pay attention to beforehand so they cannot be consistent whereas with a low-level construal, individuals initially construe concrete representations with an awareness of the details and following that with a high-level construal it is not as difficult to form global evaluations. Consequently, evaluations may not differ as much for low construal levels compared to high construal levels when individuals adopted a non-matching mindset.

Once more, an exception to this result is discerned for a low involvement / low familiarity product category. It is seen that for the e-reader case the matching hypothesis is significant for low construal levels and not significant for high construal levels. It is established that individuals are most consistent with low-level mental construals at both sessions since they use concrete details as heuristic cues for retrieval of prior evaluations. In contrast when a low-level construal is followed by a (non-matching) high-level construal, the focus of attention shifts from the concrete details to the global aspects; in which case heuristics cannot be employed. As a result consistency is reduced for this particular case.

### Theoretical and Practical Implications

One of the main theoretical contributions of this research to the literature of construal effects on preference consistency is demonstrating that high-level mental construal generates more consistent preferences compared to low-level mental construal. Even though a considerable amount of studies investigated the impact of mental construal on preference shifts, the prior literature is limited regarding research on construal effects on preference consistency. Moreover, even the limited number of studies provides conflicting results. Consequently, this finding makes a valuable contribution to research on mental construal and preference consistency.

Another important contribution of the present study is made through the measurement of preferences at two evaluation instances. Prior studies (Baskin et al., 2014; Hernandez et al., 2015; Lee et al., 2014; Liviatan et al., 2008a) generally have examined the relationship between construal levels and preferences with a between subjects design. In the current research we examined the within subjects effect of mental construal on the consistency of preferences with the temporal measurement of the consistency. This study has been the first to employ such a methodological approach within this stream of research to the best of our knowledge. The operationalization of consistency with a temporal angle makes it possible to investigate the impact of matching and non-matching construal levels on evaluations.

Furthermore, this study employs a number of different products as experimental stimuli. Therefore, the findings of the present study are not restricted to a particular item; rather evidence from different product categories corroborates each other. Besides, selecting products that the participants were less familiar and involved with put forward different effects of construal on consistency while opening the door for future research for further developments.

One major practical implication for individuals is to gain an understanding that mental construal can be used to influence consistency of one’s preferences. Once the type of mindset that creates greater consistency is known, individuals can advance methods that will enable them to become more consistent. The present research suggests that adopting a high level construal, individuals can be more consistent in their choices. For example, considering the importance of a product rather than its concrete details corresponds to high-level construal (Trope & Liberman, 2000) resulting in temporally stable choices.

Furthermore, this study provides valuable implications for both consumers and marketing practitioners. Both consumers and marketing practitioners presume consistency of preferences. From the consumers’ point of view, it is important to know that preferences won’t change rapidly; ergo, one can enjoy an item for a long while and won’t experience frequent regrets. On the other hand, from the marketing practitioners’ perspective it is equally important to be able to rely on the consistency of consumers’ preference since strategic marketing activities such as segmentation, targeting and positioning require some regularity and steadiness of preferences to be able to address them properly. Therefore, marketing communications might be used to improve the consistency of consumer preferences by controlling their construal levels. For instance, messages focusing on near vs. distant future (Khan et al., 2011; Trope & Liberman, 2000), close vs. far locations (Henderson, Fujita, Trope, & Liberman, 2006), similar vs. dissimilar people (Liviatan, Trope, & Liberman, 2008b), and high vs. low probability events (Wakslak, Trope, Liberman, & Alony, 2006) will produce lower construal levels which makes consumer preferences more susceptible. Additionally, communication content highlighting how one should use a product versus why one should use a product (Lee, Keller, & Sternthal, 2010; White, MacDonnell, & Dahl, 2011) and the consequences vs. the causes of using a product (Rim, Hansen, & Trope, 2013) will create low level construals, making it easier for the advertiser and the marketing manager to induce changes with consumer preferences.

At a broader level the implication for the practitioners is that, market leaders, who primarily seek longevity and loyalty, should try to further consistency of its customers by promoting the high-level aspects of the offerings and inducing abstract mindsets through the marketing communications. They may be better off providing abstract representation of the products, emphasizing the general appeal, and benefit of using the product. On the other hand, market followers should try to prompt shifts in preferences by providing a concrete representation and they may be better off emphasizing the peripheral features and feasibility aspects of the offering.

### Limitations and Future Directions

Current study used electronic products as experimental stimuli due to their level of complexity allowing deliberate appraisal. Future research may explore the generalizability of the findings of this study in different product categories. For instance, studies comparing the effect of mental construal on preference consistency between affect based and cognition based evaluations would shed additional light on the topic. It is demonstrated that the intensity of affect diminishes as psychological distance increase (Williams, Stein, & Galguera, 2014). Examining whether high level construal will induce more consistent preferences for hedonic products as well as utilitarian products might have essential implications.

Furthermore, even though the products of the study were selected using a pretest based on the familiarity and involvement of participants, one product, namely e-reader, scored low on these dimensions with the actual sample. The results found were in the opposite direction for this particular product category. Revealing the role of familiarity and involvement in the relationship between mental construal and preference consistency puts forward another path for future research.

This study includes tasks with high cognitive demands. It is suggested that changes in mental construal might enhance or reduce task performance based on the compatibility between mental construal and task characteristics (Förster, Friedman, & Liberman, 2004). Accordingly, even though the results of this study supports most of our predictions, testing same hypotheses using tasks that are cognitively less demanding may provide stimulating results due to the interaction between task characteristic and mental construal.

### Conclusion

Construal level theory suggests that individuals’ level of construal at the time of a product evaluation influences the type of features individuals attend to. Consequently, their preferences vary depending on their mental construal and the way they mentally illustrate products. Current study demonstrates that high level construal produces more consistent preferences than low level construal. Additionally, the effect of having matching versus non-matching construal levels at two evaluation instances, on the degree of consistency is examined. The preferences become the most consistent when participants adopted a high level construal at both evaluations.
